# Novel Barbiturate-Nitrate Compounds Inhibit the Upregulation of Matrix Metalloproteinase-9 Gene Expression in Intestinal Inflammation through a cGMP-Mediated Pathway

**DOI:** 10.3390/biom10050808

**Published:** 2020-05-25

**Authors:** Shane O’Sullivan, Jun Wang, Marek W. Radomski, John F. Gilmer, Carlos Medina

**Affiliations:** School of Pharmacy and Pharmaceutical Sciences, Trinity Biomedical Sciences Institute, Trinity College Dublin, 2 Dublin, Ireland; osullish@tcd.ie (S.O.); wangju@tcd.ie (J.W.); marek.radomski@usask.ca (M.W.R.); gilmerjf@tcd.ie (J.F.G.)

**Keywords:** inflammatory bowel disease, inflammation, NO, MMP-9, cGMP, Caco-2

## Abstract

Matrix metalloproteinase-9 is upregulated in inflammatory bowel disease. Barbiturate nitrate hybrid compounds have been designed to inhibit MMP secretion and enzyme activity. In this study, we investigated the mechanism of action of barbiturate-nitrate hybrid compounds and their component parts using models of intestinal inflammation in vitro. Cytokine-stimulated Caco-2 cells were used in all in vitro experiments. The NO donors SNAP and DETA-NONOate were used to study the effect of NO on MMP-9 mRNA. Mechanistic elucidation was carried out using the soluble guanylate cyclase (sGC) inhibitor, ODQ, and the cGMP analogue, 8-Bromo-cGMP. Further experiments were carried out to elucidate the role of NF-κB. NO donors exerted an inhibitory effect on MMP-9 mRNA in cytokine-stimulated cells. While the non-nitrate barbiturates had a limited effect on MMP-9 expression, the hybrid compounds inhibited MMP-9 expression through its NO-mimetic properties. No effect could be observed on mRNA for MMP-1 or MMP-2. The sGC inhibitior, ODQ, abolished the nitrate-barbiturate inhibition of MMP-9 gene expression, an effect which was reversed by 8-Br-cGMP. This study shows that the barbiturate scaffold is suitable for hybrid design as an MMP-9 inhibitor in cytokine-stimulated Caco-2 cells. The inhibition of MMP-9 levels was largely mediated through a reduction in its mRNA by a sGC/cGMP pathway mediated mechanism.

## 1. Introduction

The matrix metalloproteinases are a group of endopeptidases capable of digesting the extracellular matrix (ECM), basement membrane as well as having an immunomodulatory role related to activation of other proteases and inflammatory mediators [1,2,3]. Inflammatory bowel disease (IBD), which encompasses ulcerative colitis (UC) and Crohn’s disease (CD), is a chronic, relapsing condition involving inflammation of the gut leading to abdominal pain, diarrhoea, rectal bleeding and fever. MMP-9 is known to be up-regulated in IBD [4,5,6,7] and is associated with disruption of the epithelial barrier and activation of pro-inflammatory mediators [8,9,10,11]. Inhibition of this enzyme may therefore aid in reducing the severity of the disease. The development of clinically useful synthetic inhibitors has, to date, been disappointing, mainly due to dose-limiting side-effects but it is also true that the efficacy evident in animal models has not translated [12,13]. This has led to a revaluation of the precise role of specific MMPs in a given pathological setting and to investigate new strategies for modulating dysregulated MMP activity in disease tissue. Certain appropriately substituted barbiturates have been shown to possess MMP inhibitory characteristics [14,15] while being without sedative actions [16]. The barbiturates have, in general, better pharmacokinetic properties than other MMP inhibitory compounds such as, for example, the hydroxamates. Our group reported a series of barbiturate-nitrate hybrid compounds that are potent inhibitors of MMP-2 and MMP-9 at the enzyme level [17]. Incorporation of a nitrate group as a nitric oxide (NO) donor or mimetic functionality was intended to confer on the compounds an ability to modulate the enzyme levels of inducible MMP in order to complement the purely enzyme inhibitory actions of the barbiturate zinc binding group. Interactions between NO and MMP-9, which have been recently reviewed, are complex and difficult to predict [18]. In our previous work we showed that the hybrid compounds were able to reduce MMP-9 activity in cell supernatants as measured by gelatin zymography, a property that was not shared by barbiturate inhibitors not bearing a nitrate group. Furthermore, the hybrid compounds were significantly more efficacious than the non-nitrate counterparts in a model of MMP-9 dependent cancer cell invasion [17]. Consistent with this, we subsequently found that one of the hybrid compounds was more effective in an animal model of IBD than the MMP inhibitor from which it was derived or its incorporated nitrate component [19]. The objective of the present study was to determine the mechanism by which the hybrid compounds influence MMP levels, to investigate the role of NO in this and to assess the selectivity for MMP-9 over other MMP enzymes. We found that the inhibition of MMP-9 mRNA was largely mediated through a reduction in its mRNA by a sGC/cGMP-mediated pathway.

## 2. Material and Methods

All chemicals and biological materials were supplied by Sigma Aldrich^®^ (Dublin, Ireland) unless otherwise stated.

In this study, we used a group of barbiturate-nitrate hybrid compounds and their component parts that were previously synthesized in our lab [17].

### 2.1. Cell Culture

Caco-2 cells were supplied by the European Collection of Cell Cultures (ECACC, Salisbury, UK). Cells were cultured in minimum essential media (MEM) containing 20% fetal bovine serum (FBS), 1% sodium pyruvate, sodium bicarbonate 2.2 g/L, gentamicin 5 mg/L, streptomycin 10 mg/L, penicillin G 6 mg/L. Cells were maintained in a 37 °C, 5% CO_2_ humidified incubator until approximately 75% confluent. All the following in vitro experiments were carried out in FBS-free media. Cells were incubated with 3 series of test compounds at 10 µM for 30 min. The dose of 10 µM was chosen in accordance with our previous study were toxicological studies were carried out [17]. Cytokines TNF-α and IL-1β 10 ng/mL were then added and the cells were incubated for 24 h. For NO-donor experiments, solutions of SNAP or DETA-NONOate were prepared daily when needed and incubated (in the concentration range 10 to 500 µM). For co-incubation experiments with ODQ, this was added with the nitrate-barbiturate hybrids to give a concentration in the serum-free media of 10 µM as with the compounds. In experiments where 8-Br-cGMP was used, this was added with the ODQ and compounds to give a final concentration of 10 µM in the serum-free media.

### 2.2. Gelatin Zymography

The conditioned media from cell experiments was removed, centrifuged at 13,000 rpm for 5 min to remove dead cells or floating debris and the resulting cell-free supernatants were stored at −80 °C until assayed for MMP-2 and MMP-9 using gelatin zymography. Briefly, samples were normalized with respect to protein content using a Bradford assay. The enzymatic activities of MMP-2 and MMP-9 were assayed by gelatin zymography in serum-free media. The samples were electrophoresed on an SDS−PAGE containing 2% gelatin. The gels were washed with 2.5% Triton X three times for 20 min cycles. The gels were then washed twice and finally incubated with zymography buffer (0.15 M NaCl, 5 mM CaCl2, 0.05%NaN_3_, and 50 mM Tris-HCl buffer, pH 7.5) at 37 °C for 48 h. After incubation, the gels were stained with 0.025% Coomassie Brilliant Blue G250 in 25% MeOH, 10% acetic acid, and H_2_O and destained with acetic acid 8%, methanol 4%, and H_2_O. The gelatinolytic activity was detected as a band of gelatin digestion and was quantified by densitometry using gel documentation system r (Bio-Rad, Dublin, Ireland, Universalhood II and Quantity One 4.6 software) and expressed as a percentage of the positive control.

### 2.3. Quantitative PCR

Quantitative PCR (qPCR) was used to study the effect of the different compounds on expression of MMP-1, MMP-2 and MMP-9 and NF-κB in cytokine-stimulated cells. Briefly, the RNA was isolated using RNAqueous-4PCR^®^ kit from Ambion (Applied Biosystems, Waltham, MA, USA) according to the manufacturer’s protocol. The concentration and purity of the RNA yielded was measured using the NanoDrop ND-1000. RNA samples were converted to single-stranded cDNA using a High Capacity cDNA Reverse Transcription kit (Applied Biosystems, Waltham, MA, USA). As target probes, TaqMan MGB human MMP-9 (Hs 00234579_m1), NOS (Hs 01075521_m1), NFKB1 (Hs 00949904_m1), RelA (Hs 01042014_m1) and IKBKG (Hs 01006763_m1) were used. Endogenous 18s rRNA was used as a control to normalize gene expression data, and an RQ value (2^−∆∆Ct^, where Ct is the threshold cycle) was calculated for each sample. RQ values are presented as fold change in gene expression relative to the stimulated group, which was normalized to 1.

### 2.4. Nitrate and Nitrite Quantification–Modified Griess Assay

A spectrophotometric method was used to measure the nitrate and nitrite in conditioned media. Nitrate and nitrite standards were serially diluted to a range 1.6–200 µM in ddH_2_O and 200 µL of each concentration was added to 12-well plates in duplicate. Nitrate was reduced to nitrite with the addition of 200 µL saturated vanadium (III) solution (400 mg VCl_3_ in 50 mL 1M HCl) and then staining of the nitrite was carried out with rapid addition of 100 µL sulfanilamide (2% *w*/*v* in 5% *v*/*v* HCl) and 100 µL N-1-(naphthyl) ethylenediamine (NEDD) (0.1% *w*/*v* in ddH_2_O). The plate was incubated with rocking for 45 min and absorbance was read at λ 540nm. Measurement of nitrite standards were carried out as above with ddH_2_O added instead of VCL_3_ solution and ddH_2_O was used as a blank for both sets of standards. Conditioned media samples were normalised for protein concentration, and 200 µL loaded onto 12-well plates in duplicate for both methods described above used to measure the nitrite and nitrate levels. Addition of VCl_3_ to the conditioned media samples will give a measure of total NO_2_^−^ and NO_3_^−^ given NO_x_^−^.

### 2.5. NF-κB (p65) Binding Activity

The binding activity of the p65 subunit was measured using an NF-κB (p65) Enzyme Linked Immunosorbent Assay (ELISA) kit (Cayman Chemicals, Dublin, Ireland). Nuclear extraction was first carried out from cultured and treated cells after 24 h using the nuclear extraction kit (Cayman Chemicals, Dublin, Ireland) according to the manufacturer’s protocol. The biding of p65 in these nuclear extracts was then determined using the NF-κB (p65) transcription factor assay according to the manufacturer’s instructions.

### 2.6. Statistical Analysis

Analysis of results was carried out using Graph Pad Prism^®^ 5 for Windows (San Diego, CA, USA, Graph Pad software). All results shown represent *n* ≥ 3 and were analyzed using a one way ANOVA and Dunnett’s or Tukey post-test where appropriate. Graphs are presented as the mean ± the standard error of the mean (SEM) and statistical significance was judged as a *p* value of <0.05.

## 3. Results

### 3.1. Barbiturate-Nitrate Hybrids Reduce MMP-9 Expression in Cytokine-Stimulated Caco-2 Cells

**Figure 1:**
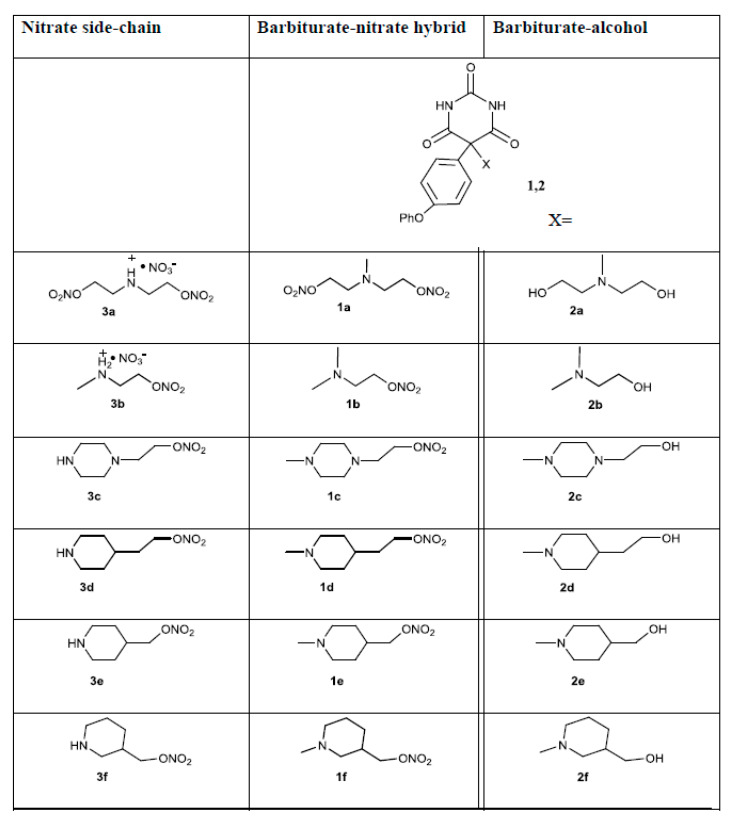
Structures of compounds used. Column one shows the nitrate side-chains. Columns two and three show the barbiturate scaffold and the nitrate and alcohol side-chains, respectively.

We have previously demonstrated that the barbiturate-nitrate hybrids (series 1, Figure 1) can reduce supernatant MMP-9 activity as measured by gelatin zymography to a greater extent than the barbiturate-alcohols (series 2, Figure 1) [17]. Here we examine the effect of the hybrid compounds at the gene level and use the barbiturate-alcohols and nitrate side-chains (series 3, Figure 1) to measure the relative contributions of the component parts of the series of compounds.

**Figure 2:**
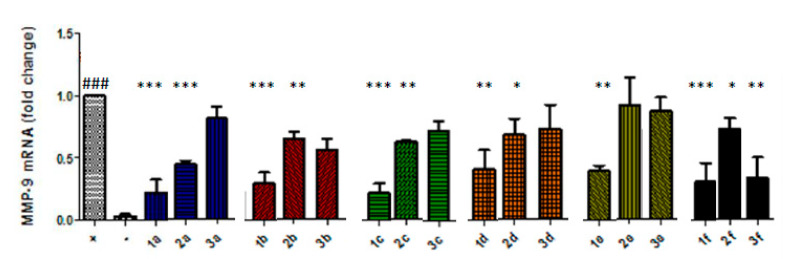
Barbiturate-nitrate hybrids (series 1) reduce MMP-9 mRNA to a greater extent than the barbiturate alcohols (series 2) or the nitrate side-chains (series 3). Caco-2 cells were incubated with series of the barbiturate-nitrate hybrids, barbiturate-alcohols or nitrate side-chains at 10 µM for 30 min prior to addition of TNF-α and IL-1β (10 ng/mL). ### *p* < 0.001 vs. unstimulated Caco-2 cells (negative control); * *p* < 0.05 vs. cytokine-stimulated Caco-2 cells (positive control); ** *p* < 0.01 vs. positive control; *** *p* < 0.001 vs. positive control.

The nitrate-barbiturates (10 µM) caused a statistically significant reduction in MMP-9 mRNA in cytokine-stimulated Caco-2 cells after 24 h compared to the untreated, stimulated cells (Figure 2). Compounds **1c** and **1a** caused the greatest mean inhibition. The alcohol-barbiturates also inhibited the transcription of MMP-9 at 10 µM but to a lesser extent. The nitrate side-chains did reduce MMP-9 expression when tested at 10 µM (Figure 2), but this inhibition did not reach statistical significance except for compound **3f**. The compounds in parallel experiments did not affect mRNA levels of MMP-1 or MMP-2, showing selectivity for inhibition of MMP-9.

### 3.2. Nitric Oxide Donors Reduce MMP-9 mRNA Levels in Cytokine-Stimulated Caco-2 Cells

In order to establish whether the effects that the nitrate-barbiturates had on MMP-9 mRNA in cytokine-stimulated Caco-2 cells were NO-mediated, we tested the effects of two NO-donors at a range of concentrations for 24 h. In this study we used the S-nitrosothiol, *S*-Nitroso-*N*-acetylpenicillamine (SNAP), which has been previously shown to have a half-life in aqueous media of approximately 4 h; the NO formation is high, where 100 μM yields about 1.4 μM NO/minute at 37 °C, and it is linear over a wide concentration range. In addition, we measured the effects of the diazeniumdiolate compound DETA-NONOate, which decomposes spontaneously and has been demonstrated to have a half-life of 20 h at pH 7.4 and 37 °C [20].

**Figure 3:**
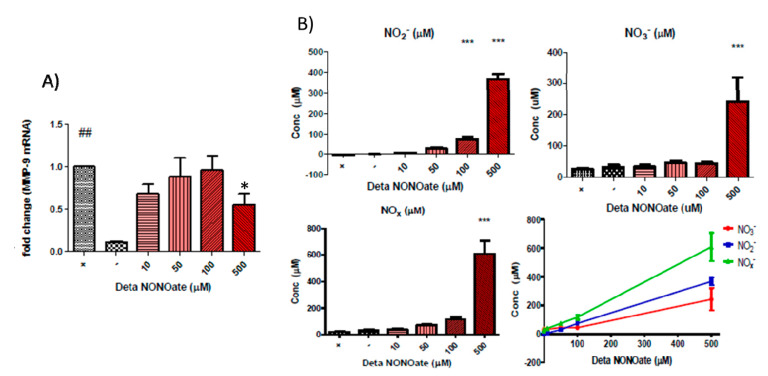
(**A**) DETA NONOate significantly reduced MMP-9 mRNA at high concentrations in cytokine-stimulated Caco-2 cells (## *p* < 0.01 vs. unstimulated Caco-2 cells (negative control); * *p* < 0.05 vs. cytokine-stimulated Caco-2 cells (positive control). (**B**) Measurements of nitrate and nitrite concentrations as breakdown products of NO by the Griess assay on the conditioned media of Caco-2 cells after 24 h of co-incubation with DETA-NONOate and pro-inflammatory cytokines TNF-α and IL-1β. Top left pane shows the nitrite concentration, top right panel shows the nitrate concentration, bottom left pane shows the combined reduced NO groups and bottom right shows the linear correlations of DETA-NONOate concentrations versus the concentration of measured NOx^−^ species (*** *p* < 0.001 vs. negative control).

We found that DETA-NONOate reduced MMP-9 expression with the highest concentration tested (Figure 3A). Figure 3B shows the release of NO species. As expected from a NONOate NO-donor, there was a linear relationship between the concentration used and the NO released with correlation analysis results for NO_x_^−^ (*p* < 0.0001, R^2^ = 0.9975), NO_2_^−^ (*p* < 0.0001, R^2^ = 0.9996) and NO_3_^−^ (*p* < 0.0001, R^2^ = 0.9828) being statistically significant. There was little to no difference in NO yielded from the lower concentrations of DETA-NONOate used, which may reflect the limited sensitivity of the Griess assay. At a concentration of 500 µM, DETA-NONOate yielded statistically significantly more of all NO_x_^−^ species than the positive control. The highest concentrations of nitrate and nitrite were produced from the highest concentration of DETA-NONOate, which also produced a significant inhibition of MMP-9 at the gene level.

**Figure 4:**
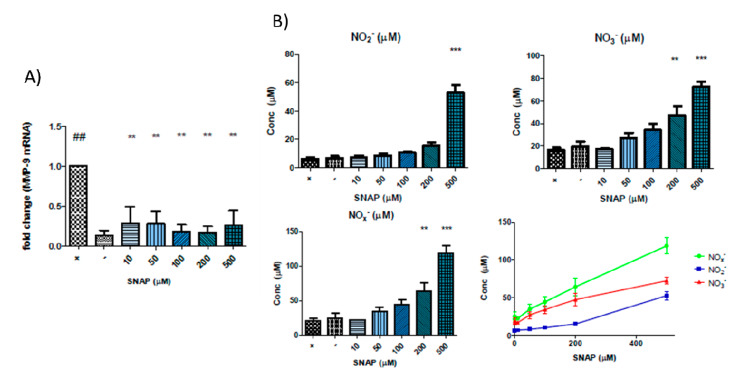
(**A**) SNAP significantly reduced MMP-9 mRNA at different concentrations tested in cytokine-stimulated Caco-2 cells: ## *p* < 0.01 vs. unstimulated Caco-2 cells (negative control); ** *p* < 0.01 vs. cytokine-stimulated Caco-2 cells (positive control). (**B**) Measurements of nitrate and nitrite concentrations as breakdown products of NO by the Griess assay on the conditioned media of Caco-2 cells after 24 h of co-incubation with SNAP and proinflammatory cytokines TNF-α and IL-1β. Top left pane shows the nitrite concentration, top right pane shows the nitrate concentration, bottom left pane shows the combined reduced NO groups and bottom right shows the linear correlations of SNAP concentrations versus the concentration of measured NOx^−^ species. (** *p* < 0.01 vs. negative control; *** *p* < 0.001 vs. negative control).

We also found that the addition of SNAP caused a significant inhibition of MMP-9 at the gene level at all concentrations tested (Figure 4A). Figure 4B shows the release of NO species. Incubations with SNAP showed a linear correlation between the concentration used and the resultant nitrate and nitrite concentrations that were present in the conditioned media after 24 h. The results of correlation analysis of SNAP concentration and NO_x_^−^ (*p* < 0.0001, r^2^ = 0.9964), NO_2_ (*p* < 0.0001, r^2^ = 0.9696) and NO_3_^−^ (*p* = 0.0002, r^2^ = 0.9525) are unsurprising considering that SNAP is expected to spontaneously yield NO, which will be decomposed to nitrite and nitrate. At 500 µM SNAP, the difference in nitrite concentrations reached statistical significance for all groups. Similar results were observed for nitrate and NO_x_^−^ concentrations, where 500 µM SNAP resulted in statistically significant differences with all other groups, and 200 µM was also statistically different from the controls and 10 µM SNAP. There is no obvious correlation between these results and the effect on MMP-9 gene expression.

### 3.3. Barbiturate-Nitrate-Hybrids Inhibit MMP-9 Gene Expression in an NF-κB-Independent Manner

In this study, we examined the effect of the barbiturate-nitrate hybrids on gene expression of various components of the NF-κB pathway and the nuclear binding of the p65 subunit to test the hypothesis that the compounds could mimic the effect of NO on NF-κB.

**Figure 5:**
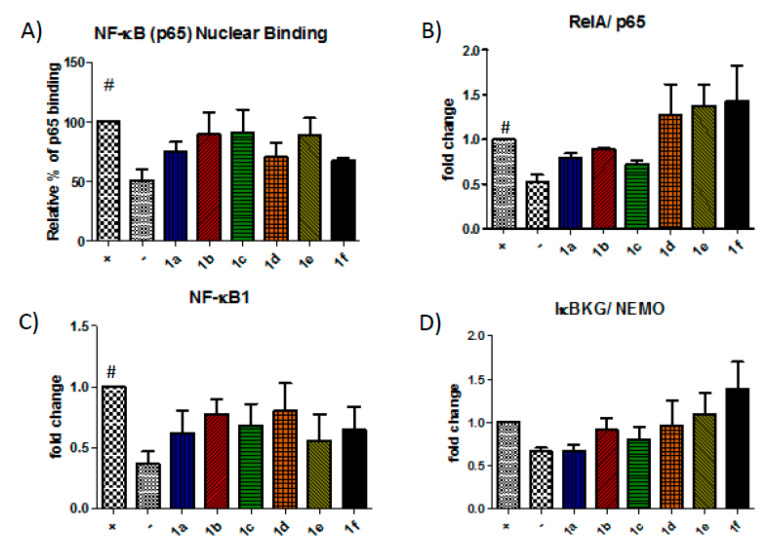
The barbiturate-nitrate hybrids show little effect on the NF-κB pathway. The nuclear translocation and binding of the NF-κB subunit p65 was measured using ELISA (**A**) and the gene expression of RelA/p65, NF-κB1/p105 and a component of the IKK complex, IκBG/NEMO were measured using qPCR (**B**–**D**). # *p* < 0.05 vs. unstimulated Caco-2 cells (negative control).

A trend towards inhibition was observed for the nitrate-barbiturates that did not reach statistical significance (Figure 5A). Therefore, we decided to examine the effect of the compounds on the transcription of some of the elements of the NF-κB pathway.

The expression of RelA/p65, NF-κB1/p105 and IκBKG/NEMO, which forms part of the IKK complex, were assessed by qPCR (Figure 5B–D). However, the compounds showed a limited effect on RelA/p65, NF-κB1/p105 and IκBKG/NEMO with **1a**, **1b** and **1c** causing the greatest reduction in expression of these elements.

### 3.4. Inhibition of MMP-9 by the Barbiturate-Nitrate Hybrids Is Partly Mediated Through a sGC/cGMP Pathway

**Figure 6:**
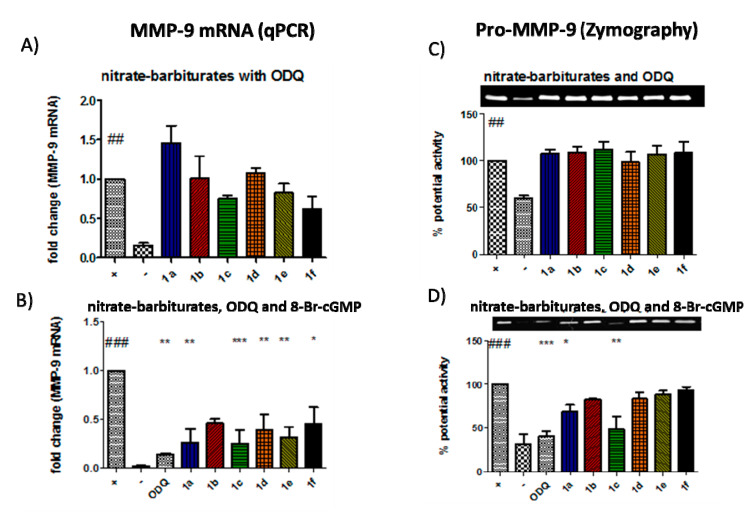
Barbiturate-nitrate hybrids inhibit MMP-9 via a soluble guanylate-cyclase-dependent pathway. (**A**) there was a significant up-regulation of the MMP-9 gene when inducing Caco-2 cells with the pro-inflammatory cytokines. When cells were activated in the presence of ODQ (0.05 μM), this resulted in a reversion of MMP-9 mRNA levels in barbiturate-nitrate hybrid-treated cells. (**B**) Exogenously added 8-bromo-cGMP restored the effect of ODQ (0.5 μM). These results correlated with the pro-MMP-9 protein activity as shown by zymography (**C**,**D**) ## *p* < 0.01 vs. unstimulated Caco-2 cells (negative control); ### *p* < 0.001 vs. negative control; * *p* < 0.05 vs. cytokine-stimulated Caco-2 cells (positive control); ** *p* < 0.01 vs. positive control; *** *p* < 0.001 vs. positive control.

Following the limited effect of the compounds in altering NF-κB nuclear binding or expression of components of the pathway, we focused on the role of the cGMP pathway in mediating the inhibition of MMP-9 by the nitrate-barbiturates. This was first achieved using the pharmacological inhibitor 1H-(1,2,4)oxadiazolo(4,3-a)quinoxalin-1-one (ODQ), which is a highly selective and irreversible heme site inhibitor of sGC and is competitive with NO [21,22]. As shown in Figure 6A, co-incubation of the nitrate-barbiturates with ODQ abolished any reduction in MMP-9 gene expression, an effect which was reversed by adding the cGMP analogue 8-Br-cGMP to the cells (Figure 6B). These effects correlated with MMP-9 protein activity as shown in Figure 6C,D.

## 4. Discussion

Understanding that MMP-9 remains an attractive target for inhibition in a variety of inflammatory conditions, our group designed a series of barbiturate-based hybrids that are intrinsically active as MMP inhibitors at the enzyme level but have additional effects on MMP activity through a nitrate moiety [17]. The present findings indicate that the hybrid compounds, which inhibit at the enzyme level, also reduce MMP-9 at the gene level in response to inflammatory stimuli. These effects are mediated by NO mimicry in cGMP activation and they are selective for MMP-9 inhibition over MMP-1 and MMP-2.

MMPIs have failed in clinical trials due to disappointing clinical efficacy results compared to animal trials and dose-limiting side-effects of MSS; but we can now reflect on how little was known of the complex protease network or the net effect of inhibition of certain enzymes in a given setting. The trials added MMPIs as co-therapies for patients with invasive or metastatic cancer, which may not have been appropriate considering that metastasis was already established and also, the genetic diversity of the disease [13]. It is now known that the MMPs may play a protective role in tumour progression [23,24] including MMP-8 and indeed much broader roles in inflammation than previously appreciated, many of which are protective [25,26]. Setting will be crucial to the success of MMPIs, and so as we understand more about the interactions of the MMP network and the roles of individual MMPs in a given disease setting, we may be better able to appropriately target them for inhibition. While broad spectrum inhibition may be appropriate in certain acute inflammatory settings, selectivity may be important in reducing side-effects in chronic inflammatory conditions. With the difficulties in finding selective and clinically useful active-site inhibitors, alternative strategies such as blocking upstream pathways of activation [27,28] or indeed targeting the transcriptional upregulation of the enzyme is of utmost importance. Xanthine-derivatives, NSAIDs and 3-hydroxy-3-methylglutaryl coenzyme A (HMG-CoA) reductase inhibitors have all been studied as inhibitors of MMP-9 expression [9]. Doxycycline, the only clinically approved drug acting through MMP inhibition [29], is a weak enzyme level inhibitor [30] which exerts its effects mainly at the transcriptional level [31,32,33].

In previous studies, we established that the hybrid compounds affect MMP-9 secretion [17], but it was unclear if this was at the storage, secretory or transcriptional level. In this study, we found a profound reduction of MMP-9 mRNA with the hybrid compounds on cytokine-stimulated cells. These effects were not observed on MMP-1 and MMP-2 mRNA. We also found a smaller but significant reduction in MMP-9 transcription with the alcohol-barbiturates. The inhibitory effect of the non-nitrates on MMP transcription is probably due to a general anti-inflammatory effect associated with MMP-9 inhibition at enzyme level. The MMPs catalyse the activation of a broad range of substrates [2,34], which can promote the transcriptional up-regulation of pro-inflammatory mediators, including MMPs themselves. MMPs have become common read-outs for inflammation in cell and animal models of IBD. For example, in a rat model of UC, the broad spectrum hydroxamate MMP inhibitor, ilomastat, was shown to inhibit MMP-1 expression in the colon [35]. The nitrate side-chains tested alone also reduced MMP-9 levels with a similar order of magnitude to the alcohol-barbiturates. The striking effect of the hybrid compounds on MMP-9 expression may be attributed to an indirect inhibitory action at the enzyme level and an additional NO-mediated effect at the mRNA level.

Next, we tested the effects of NO-donors on MMP-9 in our model of intestinal epithelial inflammation in order to ascribe some of the MMP-9 inhibitory action of the compounds to the nitrate moiety. In our experiments, SNAP and DETA-NONOate were chosen as NO-donors to represent varying NO release profiles. There was a linear relationship between the concentration of the compound used and the concentration of nitrate and nitrite, breakdown products of NO, that were in the media after 24 h. Cytokine-stimulated cells that were incubated with SNAP showed a large inhibition of MMP-9 gene expression. DETA-NONOate was used in the same range of concentrations as SNAP but did not result in the same effect on MMP-9. It is interesting to note that while there was a direct correlation between the concentration of donor used and the NO_x_^−^ species measured, this did not correlate with the effect on MMP-9 despite both NO-donors having some inhibitory effect. The half-life of NO in a biological setting is in the range of seconds and so the measurement of its breakdown products after 24 h may not necessarily be relevant. SNAP is expected to breakdown spontaneously and has a shorter half-life than DETA-NONOate and will likely produce a higher concentration of NO that may be sustained for a shorter period of time and it may be this property that resulted in the inhibition of MMP-9 mRNA.

Knowing that the nitrate-barbiturates can inhibit MMP-9 transcription, that NF-κB is involved in the upregulation of MMP-9 and that NF-κB is sensitive to NO, we decided to assess the effect of the compounds on the NF-κB pathway. NF-κB exists in the cytoplasm as an inactive dimer bound to its inhibitor IκB. Activation of the IKK complex liberates the dimer to migrate to the nucleus and interact with κB binding sites in the promoter regions of certain genes. The MMP-9 gene contains at least two of these binding sites in its promoter region [36] and NF-κB has been shown to be essential for MMP-9 upregulation [37,38,39]. NF-κB can induce the expression of iNOS when activated and NO is a known regulator of NF-κB, likely functioning in a concentration-dependent negative feedback loop [40,41]. We have previously reviewed the evidence for the NO’s modulation of NF-κB in the context of MMP-9 [18]. Therefore, we decided to assess the effect of the compounds on the NF-κB pathway. An ELISA of the p65 subunit was used to measure the nuclear binding of the NF-κB complex. There was a trend of inhibition for all the compounds with a maximum mean reduction of 32%, but the observed reductions were not statistically significant. To further investigate the effect on p65 nuclear binding, we measured the effect of the compounds on the expression of some of the components of the NF-κB pathway. RelA and NF-κB1 are common components of the NF-κB complex, a protein dimer that will bind to its response element on certain genes. Although the primary mechanism of regulation is its liberation from IκB, it is still under transcriptional control and these two components are both upregulated by pro-inflammatory cytokines in Caco-2 cells. IκBKG is part of the IKK complex, which can phosphorylate IκB, leaving the NF-κB complex free to migrate to the nucleus. The difference between the sham and stimulated controls was small, which reflects the fact that transcriptional control is not the primary mechanism of regulation of IκBKG. The compounds exerted a limited effect on RelA/p65, NF-κB1/p105 and IκBKG/NEMO.

We next turned our attention to cGMP as a candidate for the NO-mediated effect. The cGMP pathway is one of the most well-defined ways that NO exerts many of its effects such as vasodilation and inhibition of platelet aggregation. NO can react with the heme centre of sGC, increasing the rate of catalytic conversion of GTP to cGMP [42]. sGC can mediate the transcriptional upregulation of COX-2, TNF, plasminogen activator inhibitor-1 (PAI-1), vascular endothelial growth factor receptor-1 (VEGFR1), mitogen-activated protein kinase phosphatase-1 (MKP-1) and MMP-9. The exact mechanism by which cGMP exerts its transcription regulatory functions has not been fully explained but it can alter the function of cGMP-regulated ion channels, cGMP-regulated phosphodiesterases and cGMP-dependent protein kinases (PKG) [43]. Several lines of evidence suggest PKG as the mediator of sGC action on MMP-9 gene transcription [44,45,46,47]. Co-incubation of the hybrid compounds with the sGC inhibitor ODQ, abolished their MMP-9 inhibitory effects and no differences were observed between the compound treated cells and the positive control at the level of gene transcription or enzyme activity. We could therefore deduce that the nitrate-barbiturates inhibited MMP-9 transcription in a sGC-dependent manner. To confirm this result and further elucidate the pathway, the cGMP analogue, 8-Br-cGMP, was added with the compounds along with ODQ, and it was found that the inhibitory properties of the compounds were restored to what they were with the compounds alone. We can therefore conclude that nitrate-barbiturates exert their inhibitory action on MMP-9 transcription through a sGC-cGMP pathway.

In summary, both control NO donors were able to exert some inhibitory effect on MMP-9 transcription in cytokine-stimulated Caco-2 cells, but this effect was independent of the NO_x_^−^ concentration after 24 h. The barbiturate-nitrate hybrids, which are established enzyme level inhibitors, also inhibit MMP-9 at the gene level, an effect that is partly mediated by the nitrate group through a sGC-cGMP pathway. This discovery highlights the potential of these drugs in treating colonic inflammation and also represents a novel mechanism for correcting MMP dysregulation in inflammatory diseases. New studies are guaranteed to test these new compounds in other inflammatory conditions where MMP-9 upregulation plays an important role.
